# Excessive alcohol consumption: a driver of metabolic dysfunction and inflammation

**DOI:** 10.3389/ftox.2025.1670769

**Published:** 2025-09-29

**Authors:** Jaeeun Lee, Ji-Young Lee, Hyunju Kang

**Affiliations:** ^1^ Department of Nutritional Sciences, University of Connecticut, Storrs, CT, United States; ^2^ Department of Food and Nutrition, Keimyung University, Daegu, Republic of Korea

**Keywords:** alcohol, metabolic dysfunction, inflammation, alcohol-related disease, oxidative stress

## Abstract

With the increasing prevalence of alcohol-related diseases, expanding our understanding of the toxic effects of excessive alcohol consumption is critical for prevention and treatment of metabolic and inflammatory pathology. This review summarizes current knowledge on the metabolic dysfunction and inflammation caused by alcohol and their impact on the pathogenesis of alcohol-related liver disease (ALD), type 2 diabetes, cardiovascular disease, and obesity, and neurological damage. It highlights recent evidence that alcohol induces a cascade of reactive oxygen species (ROS)-mediated lipid peroxidation and nicotinamide adenine dinucleotide (NAD^+^) depletion, triggering mitochondrial dysfunction and metabolic imbalances in the liver, heart, pancreas, and brain. By integrating these mechanistic insights with emerging data on how disrupted lipid and glucose metabolism amplify immune dysregulation, the review underscores the interplay between metabolic and inflammatory pathways in exacerbating tissue injury across these organs. A deep understanding of these metabolic and inflammatory disruptions is therefore essential for developing novel therapeutic strategies, including metabolic and nutritional interventions, aimed at mitigating the health risks of excessive alcohol consumption.

## 1 Introduction

Excessive alcohol consumption is a major global health concern, responsible for an estimated 4.7% of all deaths in 2019, according to a new 2024 report from the World Health Organization (World Health Organization, 2024). It significantly contributes to the global burden of diseases, particularly liver disease, cardiovascular complications, and metabolic disorders. For the purposes of this review, “excessive alcohol consumption” refers to patterns of drinking that exceed public health guidelines, such as binge drinking (consuming four to five or more drinks on an occasion) or heavy weekly use (8–15 or more drinks per week for women and men, respectively), which can lead to health and safety risks (Crews et al., 2024; Koob, 2024). The toxicity of alcohol and its metabolites disrupts metabolic processes across multiple organs by inducing oxidative stress and depleting nicotinamide adenine dinucleotide (NAD^+^) (Tsermpini et al., 2022). Acetaldehyde, a toxic metabolite of alcohol, binds to proteins in mitochondria or microtubules, leading to structural damage and metabolic dysfunction. Furthermore, this harmful drinking pattern induces the overproduction of reactive oxygen species (ROS), culminating in oxidative stress and inflammation. This inflammatory state is a critical factor in the development of not only alcohol-related organ diseases and senescence-associated diseases but also in the pathophysiology of alcohol use disorder itself (Carvajal et al., 2023). Moreover, depletion of NAD^+^ levels due to alcohol oxidation can impair energy-generating pathways, including mitochondrial β-oxidation of fatty acids and the tricarboxylic acid (TCA) cycle, further exacerbating metabolic dysfunction (Bailey and Cunningham, 2002; Jeon and Carr, 2020).

Understanding the pathways sensitive to alcohol exposure is pivotal for the development of effective strategies to protect against alcohol-related conditions. This review summarizes the metabolic and inflammatory dysfunctions and diseases associated with excessive alcohol consumption. This exploration of alcohol-mediated dysfunctions can help identify new therapeutic approaches to protect against alcohol-related diseases.

## 2 Ethanol metabolism and its toxic effects

### 2.1 Ethanol oxidation

**FIGURE 1:**
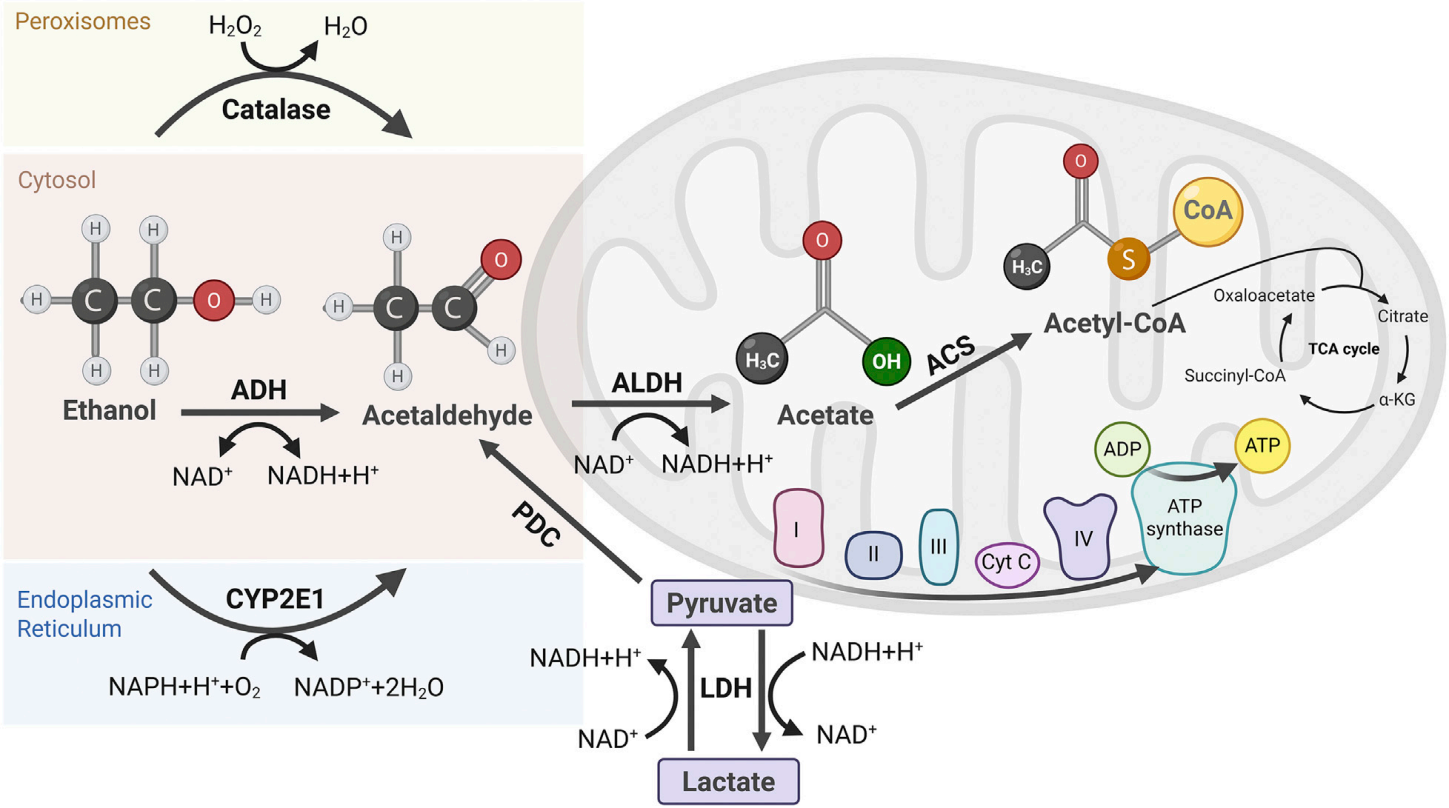
Pathways of Ethanol Metabolism and Its Byproducts. Ethanol metabolism occurs via three primary pathways. Alcohol dehydrogenase (ADH) in the cytosol oxidizes ethanol to acetaldehyde, producing NADH. Catalase in peroxisomes contributes minimally to ethanol oxidation. Cytochrome P450 2E1 (CYP2E1) in the endoplasmic reticulum (ER) generates acetaldehyde with concomitant ROS. Acetaldehyde is further oxidized to acetate by aldehyde dehydrogenase (ALDH) in mitochondria, accompanied by additional NADH generation. Acetate is then converted to acetyl-CoA by acetyl-CoA synthetase (ACS) locally or in other tissues, fueling the TCA cycle for energy production. Elevated NADH levels disrupt metabolic homeostasis, including the pyruvate-to-lactate conversion mediated by lactate dehydrogenase (LDH), altering redox balance and cellular metabolism.

The primary pathway for alcohol metabolism is the oxidation of ethanol to acetaldehyde, catalyzed by cytosolic alcohol dehydrogenase (ADH), a process requiring NAD^+^ as a cofactor (Zakhari, 2006). Acetaldehyde, a highly toxic byproduct, is further oxidized to acetate by acetaldehyde dehydrogenase (ALDH), another NAD^+^-dependent reaction (Figure 1). This sequential ethanol oxidation by ADH and ALDH occurs primarily in the cytoplasm and mitochondria of hepatocytes, respectively. Acetate, the end product of ethanol oxidation, is released into the bloodstream and transported to various peripheral tissues, where it becomes acetyl coenzyme A (acetyl-CoA) by the action of acetyl-CoA synthetase (ACS) in the mitochondria. Acetyl-CoA serves as a key intermediate in several metabolic pathways, including the TCA cycle, fatty acid synthesis, and ketone body formation, depending on the energy demands and nutritional status of the cell (Shi and Tu, 2015).

During chronic alcohol consumption or elevated blood alcohol levels, cytochrome P450 2E1 (CYP2E1), located in the endoplasmic reticulum (ER), utilizes nicotinamide adenine dinucleotide phosphate (NADPH) and oxygen to oxidize ethanol and generate ROS during this process (Lu and Cederbaum, 2008). More recent *in vivo* studies in murine models have confirmed that chronic alcohol intake upregulates CYP2E1, exacerbating oxidative liver injury (Nath et al., 2011). ROS can damage mitochondrial DNA (mtDNA) by diffusing across mitochondrial membranes or through close contact between the ER and mitochondria, leading to oxidative modifications, such as base alterations (e.g., 8-oxo-2′-deoxyguanosine), single- and double-strand breaks, and cross-linking of mtDNA proteins (Koop, 2006).

Another minor ethanol oxidative pathway is present in peroxisomes, where ethanol is metabolized by catalase, especially in conditions of elevated hydrogen peroxide, often associated with chronic alcohol consumption (Zakhari, 2006). Also, non-oxidative ethanol metabolism occurs at the cellular level, although it is relatively minor. Fatty acid ethyl ester synthases catalyze the reaction between ethanol and fatty acids to form fatty acid ethyl esters (FAEEs), which can disrupt membrane integrity, induce inflammatory responses, and interfere with mitochondrial function (Lee et al., 2021). Although the accumulation of FAEEs exacerbates liver inflammation and fibrosis, key features of alcoholic steatosis (Park et al., 2023), their overall contribution to alcohol metabolism is considerably insignificant compared to the oxidative pathways (Figure 1).

Excessive alcohol consumption results in cellular and systemic toxicity driven by acetaldehyde and ROS, which disrupt cellular processes, leading to oxidative stress, inflammation, and mitochondrial dysfunction. This cascade compromises cellular integrity and contributes to the progression of alcohol-related diseases (ALDs) through widespread tissue and organ damage.

### 2.2 Oxidative stress and cellular damage

A primary mechanism of alcohol-induced toxicity is the excessive generation of ROS during its metabolism. Ethanol is oxidized by ADH and CYP2E1 into acetaldehyde, which is subsequently converted into acetate by ALDH (Zakhari, 2006). This process generates large amounts of ROS, overwhelming the cell’s antioxidant defenses and triggering oxidative stress (Beckhauser et al., 2016). The accumulation of ROS causes oxidative damage of lipids, proteins, and DNA, resulting in cellular dysfunction and tissue injury (Juan et al., 2021).

The excessive production of ROS also depletes NAD^+^, disrupting the balance of the NAD^+^/NADH ratio, which is essential for cellular energy production and metabolic processes. Furthermore, *in vitro* cell culture studies have shown that ROS accumulation also activates enzymes such as NADPH oxidase-2 and cyclooxygenase-2, further exacerbating oxidative damage and cellular injury (Bedard and Krause, 2007; Onodera et al., 2015). Additionally, alcohol impairs cells’ antioxidant defenses, particularly by depleting the levels of glutathione, a key antioxidant that neutralizes ROS (Huang et al., 2009). This impairment exacerbates oxidative damage across major organ systems, such as the liver, brain, and cardiovascular system.

Another critical consequence of alcohol-induced oxidative stress is the repression of sirtuin 1 (SIRT1) activity, an NAD^+^-dependent deacetylase, which regulates inflammation and oxidative stress responses (Singh and Ubaid, 2020; Tsermpini et al., 2022). Indeed, an *in vitro* study using RAW 264.7 macrophages linked reduced SIRT1 activity by ethanol exposure to increased inflammation and metabolic dysfunction (Kang et al., 2021a). Taken together, these interconnected mechanisms, including ROS overproduction, NAD^+^ depletion, and SIRT1 repression, play a significant role in alcohol-related cellular and systemic damage.

The liver is the central organ responsible for most ethanol metabolism, primarily by ADH and CYP2E1, decreasing the NAD^+^/NADH ratio (Cederbaum, 2012). Elevated NADH levels inhibit fatty acid oxidation and promote lipogenesis by increasing levels of acetyl-CoA and malonyl-CoA (Cortés-Rojo et al., 2020). Malonyl-CoA further inhibits carnitine palmitoyltransferase 1, blocking the transport and oxidation of fatty acids in mitochondria, leading to lipid accumulation and steatosis (Dabravolski et al., 2021). Also, ROS generated by CYP2E1 further intensifies oxidative stress, lipid peroxidation, and inflammation (Ali et al., 2019), contributing to the progression of ALD from simple steatosis to alcoholic hepatitis and cirrhosis. Chronic alcohol consumption triggers progressive cycles of liver injury, repair, and scarring, ultimately leading to hepatic fibrosis, cirrhosis, and liver failure. The toxic byproducts of ethanol metabolism, such as acetaldehyde and ROS, damage liver cells and impair their ability to detoxify harmful substances, such as endotoxins and gut-derived bacteria (Osna et al., 2017). As liver cells are exposed to sustained damage, their detoxifying capacity decreases, exacerbating systemic toxicity (Osna et al., 2017).

### 2.3 Inflammation

Alcohol-induced oxidative stress triggers an inflammatory response in the liver, the primary site of alcohol metabolism and inflammation (Shukla et al., 2021). The breakdown of alcohol generates ROS and pro-inflammatory mediators, including cytokines and chemokines, which contribute to tissue damage and disease progression (Wheeler, 2003). In the liver, Kupffer cells (resident macrophages) play a central role in mediating alcohol-induced inflammation. Gut-derived lipopolysaccharides (LPS) translocate to the liver due to increased intestinal permeability, activating toll-like receptor 4 (TLR4) on Kupffer cells. This activation proceeds via the MyD88-dependent signaling pathway, which culminates in the activation of the transcription factor nuclear factor kappa B (NF-κB). Activated NF-κB then translocates to the nucleus and promotes the transcription of pro-inflammatory cytokines, including tumor necrosis factor (TNF) and interleukin-6 (IL-6) (Su et al., 2000). This chronic inflammatory state, driven by cytokines and ROS, promotes the progression of ALD, fibrosis, and cirrhosis. Alcohol also activates the NOD-like receptor family pyrin domain containing 3 (NLRP3) inflammasome complex within these immune cells. This activation is triggered by endogenous signals stemming directly from alcohol-induced cellular stress, such as mitochondrial ROS overproduction and the release of damaged mitochondrial DNA (mtDNA) into the cytosol. The assembled NLRP3 inflammasome activates caspase-1, which in turn cleaves pro-interleukin-1β (pro-IL-1β) into its mature, highly inflammatory form, IL-1β, a critical mediator in ALD pathogenesis (Kelley et al., 2019).

Beyond the liver, ethanol metabolism significantly affects the gastrointestinal (GI) tract by disrupting the intestinal barrier and altering the gut microbiome. Although the liver is the primary site for ethanol metabolism, enzymes such as ADH and ALDH are also present in the intestinal mucosa (Jelski et al., 2014). Intestinal bacteria further contribute to ethanol metabolism, producing additional acetaldehyde, which damages intestinal cells by forming adducts with proteins and DNA. Both the small and large intestines are affected by these changes, leading to chronic inflammation, disruption of tissue homeostasis, and eventual organ dysfunction (Patel et al., 2015). Chronic ethanol consumption also results in gut dysbiosis, an imbalance between beneficial and pathogenic bacteria (Litwinowicz et al., 2020). This imbalance, coupled with excessive acetaldehyde production, exacerbates inflammation in the GI tract. As ethanol metabolism progresses, tight junction proteins such as claudins and occludins are downregulated, increasing the permeability of the intestinal mucosa, commonly known as a “leaky gut”. This increased permeability allows toxins and bacterial products, such as LPS, to enter the bloodstream, triggering systemic inflammation (Bishehsari et al., 2017). Recent *in vivo* animal studies have further established this mechanism also disrupts mucosal immune defenses, suppressing Paneth cells that produce antibacterial compounds, leading to bacterial overgrowth, which intensifies inflammatory responses in the intestine and liver (Zhong et al., 2020).

Alcohol further disrupts the gut barrier by reducing protective molecules like intestinal trefoil factor and increasing ROS production, which weakens the epithelial lining (Morris and Yeligar, 2018). Moreover, alcohol reduces the production of short-chain fatty acids (SCFAs) (Litwinowicz et al., 2020), including butyrate, which exacerbates gut permeability and systemic inflammation, linking alcohol consumption to diseases in other organs, particularly the liver (Fairfield and Schnabl, 2021). This allows harmful substances to infiltrate the bloodstream, amplifying systemic inflammation. Additionally, chronic alcohol exposure impairs vitamin metabolism, such as thiamine (vitamin B1), leading to deficiencies such as Wernicke-Korsakoff syndrome, which causes cognitive impairment (Martin et al., 2003). These interconnected mechanisms underscore the significant impact of alcohol on the gut-liver axis and its contribution to the progression of ALD and other alcohol-induced pathologies.

### 2.4 NAD^+^ metabolism and cellular energy disruption

NAD^+^ is required for ethanol oxidation by ADH and ALDH, but its continuous consumption during alcohol metabolism depletes cellular levels, disrupting redox balance and impairing other NAD^+^-dependent functions. NADH produced by ADH is transported into mitochondria through the α-glycerophosphate or malate-aspartate shuttles for re-oxidation (Lu et al., 2008; Cederbaum, 2012), a process that supports NAD^+^ regeneration (Xie et al., 2020). The efficiency of these transporters and the capacity of the mitochondrial respiratory chain to oxidize NADH determine the rate of ethanol oxidation and the cell’s ability to maintain redox homeostasis. We reported that alcohol depletes the cellular NAD^+^ pool and inhibits its synthesis. Alcohol inhibits the expression of nicotinamide phosphoribosyltransferase (NAMPT), the rate-limiting enzyme of the NAD^+^ salvage pathway, which was counteracted by an NAD^+^ precursor or a SIRT1 activator in ethanol-stimulated macrophages (Kang et al., 2021a; Kang et al., 2021b; Gwon et al., 2024). This inhibition, due to chronic alcohol consumption, exacerbates NAD^+^ deficiency in the liver and macrophages, further impairing metabolic functions, including fatty acid oxidation, glycolysis, and mitochondrial respiration, intensifying the metabolic imbalance and contributing to disease progression (Kang et al., 2022; Kang et al., 2020).

The reduction in NAD^+^ also hinders the activity of SIRT1, which relies on NAD^+^ as a cofactor (Zhang et al., 2009). As SIRT1 activity declines, the regulation of key metabolic processes, such as DNA repair, oxidative stress response, and inflammation control, is disrupted, further intensifying the cellular damage caused by alcohol. This dysfunction contributes to increased oxidative stress, greater DNA damage, and chronic inflammation associated with long-term alcohol exposure (Iside et al., 2020).

### 2.5 Mitochondrial dysfunction and dynamics

Mitochondria are crucial organelles responsible for energy production through oxidative phosphorylation. Alcohol metabolism severely impacts mitochondrial function by disrupting the NAD^+^/NADH balance and generating excessive ROS. Chronic alcohol consumption impairs mitochondrial oxidative phosphorylation, reducing ATP production and compromising the cell’s ability to generate energy efficiently (Hoek et al., 2002). Excessive ROS production in mitochondria damages mtDNA, proteins, and lipids, leading to impaired mitochondrial function. These oxidative modifications contribute to lipid peroxidation, protein dysfunction, and mtDNA mutations, further reducing mitochondrial energy capacity (Guo et al., 2013). Alcohol also disrupts mitochondrial dynamics, which regulate fission and fusion processes (Palma et al., 2020). Under normal conditions, these processes maintain mitochondrial health by removing damaged mitochondria through mitophagy and promoting content exchange to ensure functionality. However, alcohol promotes excessive mitochondrial fission and inhibits fusion, leading to mitochondrial fragmentation and impaired mitophagy (Palma et al., 2020; Siggins et al., 2023). Fragmented mitochondria are less efficient at energy production and more vulnerable to oxidative stress, which accelerates the decline in mitochondrial function (Liu et al., 2020).

Our recent findings from an *in vitro* study in Kupffer cells indicate that chronic ethanol exposure activates the mitochondrial unfolded protein response (UPR^mt^), an adaptive mechanism designed to manage the accumulation of misfolded proteins within mitochondria (Lee et al., 2025). Under normal conditions, UPR^mt^ upregulates molecular chaperones and proteases to restore protein homeostasis (Pellegrino et al., 2013). However, prolonged ethanol exposure overwhelms this system, leading to further mitochondrial dysfunction. Notably, ethanol increases the nuclear translocation of activating transcription factor 5 and upregulates UPR^mt^-related genes in mouse Kupffer cells concomitantly with increased mitochondrial dysfunction.

## 3 Pathogenesis of alcohol-induced diseases

**FIGURE 2:**
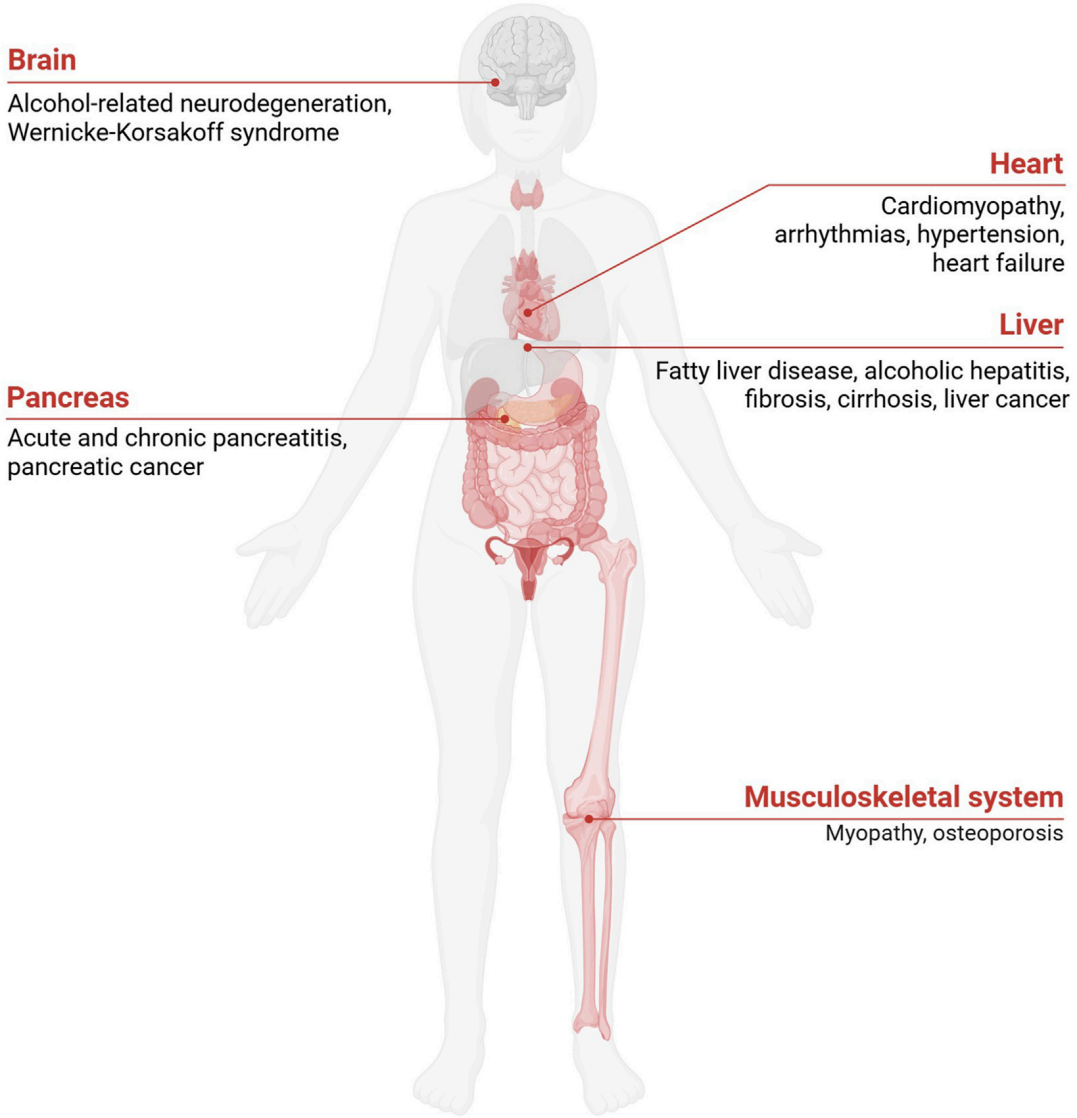
Pathological Consequences of Excessive Alcohol Consumption. Chronic alcohol consumption impacts multiple organ systems, including the brain, heart, liver, stomach, pancreas, kidney, musculoskeletal system, and reproductive system. Excessive alcohol consumption also promotes systemic inflammation and fat accumulation, exacerbating metabolic dysfunctions and increasing the risk of organ-specific diseases.

**FIGURE 3:**
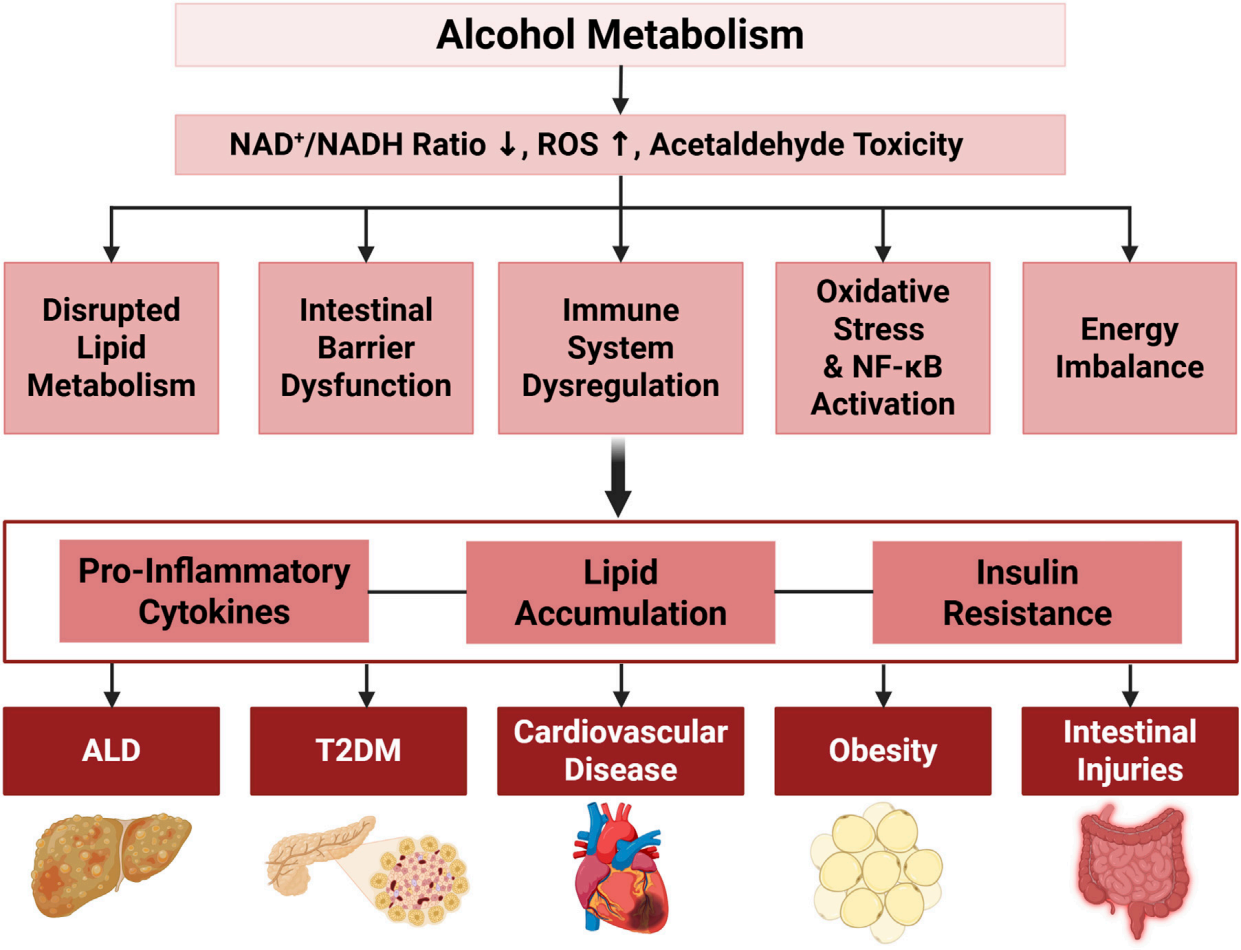
The Effects of Alcohol on Metabolic and Inflammatory Pathways Associated with Various Disorders. Alcohol metabolism disrupts cellular homeostasis by decreasing the NAD^+^/NADH ratio, increasing ROS, and inducing cellular toxicity, leading to disrupted lipid metabolism, intestinal microbiota and barrier function alterations, immune system dysregulation, oxidative stress with nuclear factor kappa-light-chain-enhancer of activated B cells (NF-κB) activation, and energy imbalance through ADH1B activity. Key downstream effects include lipid accumulation and reduced β-oxidation, loss of beneficial gut bacteria with increased ROS, and elevated pro-inflammatory cytokines (e.g., Chemokine (C-C motif) ligand 2 (CCL2), IL-6, TNFα, IL-1β, IL-8). These processes contribute to obesity, lipid/glucose imbalances, and systemic inflammation, leading to the pathogenesis of ALD, T2DM, cardiovascular disease, obesity, and intestinal injuries.

Excessive alcohol consumption disrupts metabolic processes far beyond the liver, leading to systemic dysfunctions that affect various organs. This disruption is primarily associated with lipid and glucose metabolism, resulting in metabolic imbalances that elevate the risk of conditions such as type 2 diabetes mellitus (T2DM), cardiovascular diseases (CVDs), and obesity. Alcohol’s impact on these pathways impairs insulin sensitivity, lipid metabolism, and energy balance, collectively driving the development and progression of these diseases. Figure 2 summarizes the systemic effects of chronic ethanol consumption, depicting its impact on multiple organ systems, including the brain, liver, pancreas, and musculoskeletal system, and its association with various diseases. Figure 3 presents the role of alcohol metabolism in metabolic and inflammatory disorders, detailing the disruption of lipid metabolism, intestinal barrier integrity, immune homeostasis, oxidative stress, and their downstream consequences leading to conditions such as obesity, T2DM, and ALD.

### 3.1 Alcohol-related liver disease (ALD)

ALD is a major cause of alcohol-related mortality and encompasses a spectrum of liver conditions, ranging from alcoholic fatty liver (AFL) to cirrhosis and hepatocellular carcinoma (HCC) (Figure 2) (Prince et al., 2023). The progression of ALD is driven by chronic inflammation, oxidative stress, and metabolic dysfunction triggered by excessive alcohol consumption. The earliest stage of ALD is AFL, characterized by hepatic steatosis, or fat accumulation within hepatocytes (Yan et al., 2023). This stage can progress to alcoholic steatohepatitis, marked by liver inflammation, hepatocyte injury, and ballooning degeneration. Prolonged alcohol intake promotes persistent inflammation and fibrosis, eventually leading to cirrhosis, which significantly increases the risk of HCC (Fu et al., 2024).

Alcohol consumption triggers the activation of the NLRP3 inflammasome in Kupffer cells, which in turn activates caspase-1 and induces the release of IL-1β, exacerbating inflammation and liver damage (Brahadeeswaran et al., 2023). Additionally, alcohol-induced ROS production via CYP2E1 leads to mitochondrial dysfunction and oxidative stress, compromising liver regeneration and contributing to hepatocyte damage (Jin et al., 2013). Reactive nitrogen species (RNS), particularly peroxynitrite, further contribute to liver damage through nitrosative stress (Abdelmegeed and Song, 2014). Alcohol-induced disruptions in lipid metabolism also play a pivotal role in developing ALD by altering the NAD^+^/NADH ratio and impairing mitochondrial β-oxidation of fatty acids, which causes triglyceride accumulation in the liver (Figure 3) (Jeon and Carr, 2020). This metabolic imbalance is exacerbated by increased expression of sterol regulatory element-binding protein 1, a key regulator of lipid biosynthesis, and reduced activity of PPARα, a transcription factor essential for fatty acid oxidation (Hu et al., 2012). Moreover, alcohol-induced gut dysbiosis and increased intestinal permeability allow bacterial endotoxins to enter the liver, amplifying inflammation and hepatic damage (Bishehsari et al., 2017).

These intersecting mechanisms highlight the severe, multi-faceted nature of ALD. While alcohol moderation is often discussed as a preventive measure, substantial evidence indicates that toxic effects are present even at low to moderate levels of consumption (Cui and Koob, 2017; Eckardt et al., 1998). Furthermore, liver damage such as mitochondrial dysfunction can persist even after extended periods of abstinence, suggesting that recovery is neither immediate nor guaranteed (Salazar et al., 2025). Therefore, the most effective strategy to prevent the initiation and progression of ALD is the avoidance of alcohol. For individuals with existing damage, therapeutic approaches aimed at mitigating oxidative stress and cellular injury are being actively investigated (Wagner et al., 2024).

### 3.2 Type 2 diabetes mellitus (T2DM)

Excessive alcohol consumption is a known contributor to both the onset and progression of T2DM due to its detrimental impacts on insulin secretion and sensitivity. Alcohol impairs pancreatic β-cell function, reducing basal insulin secretion and increasing β-cell apoptosis (Steiner et al., 2015; Lin and Sun, 2010). This β-cell dysfunction, combined with alcohol-induced insulin resistance in the liver and skeletal muscle, disrupts glucose homeostasis, a hallmark of early-stage T2DM (Phielix and Mensink, 2008). Alcohol also interferes with glycogenolysis and gluconeogenesis, essential for maintaining blood glucose levels, which increases the risk of abnormal blood glucose regulation and hypoglycemia (Mauvais-Jarvis and Kahn, 2000; Kim and Kim, 2012). In the liver, alcohol activates hypoxia-inducible factor 1 (HIF-1), which upregulates glucose transporter 1, leading to increased glucose uptake and potential glucose toxicity (Morris and Yeligar, 2018; Nath et al., 2011).

Additionally, alcohol-induced dysregulation of key metabolic hormones, such as ghrelin and leptin, further contributes to the pathogenesis of T2DM. Ghrelin, a hormone that stimulates insulin secretion, is disrupted in T2DM patients who consume alcohol excessively, leading to increased hepatic glucose production and reduced peripheral glucose utilization (Kim and Kim, 2012). Leptin, which normally suppresses ghrelin and regulates glucose metabolism, is also dysregulated, exacerbating insulin resistance and glucose intolerance (Ju et al., 2011). Ethanol and its toxic metabolites accumulate in pancreatic tissues, causing cellular damage and inflammation, potentially leading to acute and chronic pancreatitis. The production of FAEEs during non-oxidative ethanol metabolism is particularly toxic to pancreatic cells, disrupting cellular membranes, promoting calcium overload, and inducing cell death, further contributing to alcoholic pancreatitis (Chowdhury and Gupta, 2006; Lee et al., 2021). Reduced insulin production diminishes the ability of the pancreas to maintain glucose homeostasis, worsening systemic effects such as hyperglycemia and insulin resistance, which exacerbate the metabolic complications of chronic alcohol consumption (Kim et al., 2010).

Taken together, these mechanisms indicate that excessive alcohol consumption accelerates both the development and severity of T2DM by impairing insulin function, altering glucose metabolism, and disrupting key metabolic hormones. These combined mechanisms underscore alcohol’s significant role in promoting T2DM progression and complications.

### 3.3 Cardiovascular disease (CVD)

The relationship between alcohol consumption and cardiovascular health is complex. Moderate alcohol intake has been associated with protective effects, including increased high-density lipoprotein (HDL) cholesterol levels, improved endothelial function, and reduced platelet aggregation that may lower the risk of coronary artery disease and atherosclerosis (Krenz and Korthuis, 2012; Perissinotto et al., 2010). However, these potential benefits are not attributed to all alcoholic beverages but are primarily linked to moderate red wine consumption, largely due to its high content of beneficial polyphenols (Castaldo et al., 2019). Furthermore, this association is often observed in the context of broader healthy lifestyle patterns, such as the Mediterranean diet, making it difficult to isolate the effects of red wine alone (Santos-Buelga et al., 2021). The evidence remains debated, and further research is required to fully elucidate the specific cardiovascular benefits of red wine and its components (Martínez-González and Hernández Hernández, 2024).

In contrast, excessive alcohol intake significantly elevates the risk of CVDs, such as alcohol-induced cardiomyopathy, characterized by reduced myocardial contractility, cardiac hypertrophy, and interstitial fibrosis (Figure 2) (Husain et al., 2014; Rehm et al., 2017). Chronic alcohol consumption disrupts lipid metabolism, raising low-density lipoprotein cholesterol and triglyceride levels, thereby contributing to atherosclerosis and hypertension (Husain et al., 2014). Additionally, excessive alcohol consumption elevates blood pressure and promotes vascular endothelial dysfunction, oxidative stress, and inflammation, all of which contribute to the development of CVDs (O'Keefe et al., 2018). The production of acetaldehyde and ROS in cardiac myocytes induces oxidative stress, mitochondrial dysfunction, and inflammation. These processes compromise structural and functional integrity of heart cells, impairing ATP production, disrupting calcium handling, and triggering apoptosis (El-Mas and Abdel-Rahman, 2019).

In addition, ethanol metabolism in the heart alters lipid metabolism, promoting dyslipidemia and fat accumulation within cardiac tissues by interfering with the regulation of lipid-modulating transcription factors, such as peroxisome proliferator-activated receptors (PPARs) (El-Mas and Abdel-Rahman, 2019). This lipid imbalance, along with the oxidative damage, accelerates the development of atherosclerosis and coronary artery disease. Chronic alcohol consumption also raises circulating catecholamine levels, leading to vasoconstriction and increased blood pressure, which heightens the risk of hypertension, stroke, and other cardiovascular complications (Hillbom et al., 2011). Cardiac dysrhythmias, such as atrial fibrillation, are also common in individuals who consume alcohol excessively, further compounding their cardiovascular risk (Husain et al., 2014).

Several genetic factors can influence individual susceptibility to alcohol-related cardiovascular damage. Variants of ADH and ALDH, such as ADH1B and ALDH2*2, alter the metabolism of alcohol and acetaldehyde, potentially increasing cardiovascular risks in certain populations (Chen et al., 2020). In summary, while moderate alcohol consumption may provide certain cardiovascular benefits, excessive intake overwhelmingly disrupts cardiovascular health, underscoring the critical balance needed to prevent alcohol-induced CVD.

### 3.4 Obesity and malnutrition

Alcohol’s high caloric content (7 kcal/g) can contribute to weight gain and obesity, especially in heavy drinkers. Beyond caloric intake, alcohol affects several metabolic pathways that promote fat accumulation, particularly in the liver and other peripheral tissues (Boyle et al., 2018; Ruhl and Everhart, 2005). Chronic alcohol consumption enhances lipolysis in adipose tissue, increasing circulating free fatty acids (FFAs), which are subsequently stored in the liver as triglycerides, contributing to alcoholic fatty liver disease (Li and Ding, 2017; Steiner and Lang, 2017). Ethanol metabolism significantly impacts adipose tissue by increasing lipolysis, altering hormone regulation, and promoting insulin resistance, leading to greater release of FFAs into the bloodstream (Mathur et al., 2023). Alcohol also disrupts the regulation of hormones involved in fat metabolism, such as adiponectin, leptin, and resistin (He et al., 2015). A primary target of this disruption is adiponectin, a hormone that promotes fatty acid oxidation, exerts anti-inflammatory effects, and improves insulin sensitivity (Xu et al., 2003). By suppressing the secretion and reducing plasma levels of adiponectin, alcohol triggers a cascade of metabolic dysfunctions, including impaired glucose tolerance, insulin resistance, and elevated circulating FFAs, which collectively exacerbate hepatic lipotoxicity and worsen obesity-related metabolic disorders (Steiner and Lang, 2017). Alcohol-induced upregulation of the microsomal ethanol-oxidizing system (MEOS), primarily involving CYP2E1, reduces fat utilization, favoring fat storage and further increasing the risk of obesity (Jiang et al., 2020; Suter et al., 2014).

Conversely, significant weight loss and malnutrition are also frequently observed, particularly in individuals with severe alcohol use disorder. This paradox is often linked to alcohol-associated disordered eating patterns, where alcoholic calories substitute for nutritious food. This disruption primarily occurs at the brush border membrane of the small intestine, where alcohol alters the transport of nutrients like glucose, amino acids, vitamins, and minerals (Butts et al., 2023). Ethanol disrupts the structural integrity of intestinal cells, reduces villus height, and affects transporter proteins, leading to increased intestinal permeability and decreased absorption efficiency (Elamin et al., 2014). This compromised intestinal barrier not only leads to malabsorption but also exacerbates systemic inflammation by allowing bacterial endotoxins to translocate into the bloodstream, further driving metabolic dysfunction. Additionally, alcohol’s low nutritional value and substitution for food contribute to deficiencies, cellular damage, and metabolic imbalances (Kokavec, 2008). Chronic alcohol consumption can also modulate the hypothalamic-pituitary-adrenal axis, affecting hunger signals and reducing appetite, particularly for carbohydrate-rich foods (Inder et al., 1995; Kokavec, 2008). These combined effects impair digestion and promote a negative energy balance, exacerbating malnutrition and health complications (Bode and Bode, 1997).

Genetic factors can also play a role, as evidenced by the ADH1B gene variant (rs1229984), which has been linked to significant weight gain in individuals who consumed alcohol, further highlighting the genetic susceptibility to alcohol-induced obesity (Yokoyama et al., 2013). Taken together, these findings highlight alcohol’s paradoxical and damaging role in energy balance, contributing to obesity in some while causing severe malnutrition in others, often in the context of disordered eating. Therefore, limiting alcohol consumption is crucial for reducing both health risks and its associated metabolic complications.

### 3.5 Neurological damage

Ethanol metabolism significantly affects the brain as alcohol can cross the blood-brain barrier (Wu and Cederbaum, 2003) and be metabolized locally (Figure 2). Acetaldehyde exerts neurotoxic effects, such as impaired synaptic function and neuronal apoptosis, by forming adducts with proteins and DNA that disrupt cellular processes (Takeuchi and Saito, 2005; Nutt et al., 2021). Chronic ethanol exposure disrupts brain energy metabolism, leading to reduced glucose uptake and impaired mitochondrial function in neurons. These energy deficits, along with excessive ROS production, contribute to neurodegenerative conditions, including alcohol-related dementia and Wernicke-Korsakoff syndrome (León et al., 2021; Sanvisens et al., 2017).

Beyond direct toxicity from its metabolites, a central mechanism of alcohol-induced brain injury is the induction of a persistent neuroinflammatory state. While chronic alcohol use is known to activate microglia, it also triggers a reactive response in other glial cells like astrocytes. This widespread glial activation results in the release of a barrage of pro-inflammatory cytokines (e.g., TNF-α, IL-1β), chemokines, and additional ROS within the brain. This sustained neuroinflammation, driven by oxidative stress, damages neurons, disrupts synaptic plasticity, and is now understood to be a key driver of the cognitive deficits and emotional dysregulation characteristic of alcohol use disorder (Pervin and Stephen, 2021).

Moreover, alcohol disrupts neurotransmitter balance, with effects varying by brain region and neuronal cell type, by enhancing gamma-aminobutyric acid (GABA) activity and inhibiting glutamate signaling, leading to impairments in mood regulation, memory, and motor coordination (Oscar-Berman and Marinkovic, 2003). Over time, the combined insults of direct toxicity, oxidative stress, persistent neuroinflammation, and widespread neurotransmitter dysregulation impair synaptic plasticity, reduce cognitive function, and lead to significant neuronal loss and brain atrophy (León et al., 2021).

### 3.6 Alcoholic myopathy and muscle wasting

In addition to the organ systems discussed, ethanol exerts adverse effects on skeletal muscle by suppressing the mammalian target of rapamycin (mTOR) signaling, leading to reduced muscle protein synthesis (Levitt et al., 2022) and activating the ubiquitin-proteasome pathway for muscle protein degradation (Preedy et al., 2002). Additionally, its metabolites, e.g., acetaldehyde and acetate, further disrupt glucose and fatty acid metabolism (Steiner and Lang, 2015). In skeletal muscle, acetate derived from ethanol metabolism is converted into acetyl-CoA and enters the TCA cycle for energy production (Wilson and Matschinsky, 2020).

Chronic alcohol consumption reduces insulin sensitivity by impairing the activation of key proteins in insulin signaling, such as insulin receptor substrate-1 (IRS-1) and AKT, leading to reduced glucose uptake in muscle cells (Steiner et al., 2015). This disruption is further exacerbated by oxidative stress and pro-inflammatory cytokines, which interfere with normal insulin signal transduction and promote insulin resistance. The resulting decrease in glucose utilization leads to muscle wasting, a hallmark of alcoholic myopathy, characterized by muscle weakness, atrophy, and loss of muscle mass (Simon et al., 2017). Chronic alcohol exposure also inhibits protein synthesis by suppressing the mTOR pathway and increases protein degradation through the activation of the ubiquitin-proteasome system, involving muscle-specific E3 ubiquitin ligases, such as muscle ring finger 1 and atrogin-1, leading to muscle wasting (Simon et al., 2017). In addition, increased production of ROS in muscle, often driven by impaired mitochondrial function by chronic alcohol exposure, further damages muscle fibers and reduces ATP production, limiting muscle endurance and performance and causing structural damage, particularly in fast-twitch muscle fibers (Powers, 2014).

## 4 Summary and future directions

This review has highlighted the extensive detrimental effects of excessive alcohol consumption across multiple organ systems. These harmful effects stem from alcohol’s toxicity, its promotion of oxidative stress, and the substantial reduction in the NAD^+^/NADH ratio. Alcohol and its metabolites disturb the regulation of lipid and glucose metabolism, increasing the production of ROS and RNS, which further drives oxidative stress and inflammation, ultimately leading to cellular and organ damage. These interconnected pathways not only drive a range of chronic diseases, including liver disease, CVD, T2DM, and obesity, but also provide a clear roadmap for developing targeted therapeutic strategies.

While abstinence is paramount, emerging approaches aim to target the core mechanisms of injury. Promising pharmacological strategies include blocking inflammatory cell recruitment with chemokine receptor antagonists. Furthermore, nutritional and metabolic interventions counteract alcohol-induced damage. Supplementation with NAD^+^ precursors like nicotinamide riboside (NR) or nicotinamide mononucleotide (NMN) helps replenish the depleted NAD^+^ pool, while therapies targeting the gut-liver axis, such as probiotics or fecal microbiota transplantation work to repair intestinal barrier function and reduce systemic inflammation.

Ultimately, the shared mechanisms of oxidative stress, inflammatory responses, mitochondrial dysfunction, and metabolic disruption demonstrate alcohol’s systemic impact. This mechanistic overlap underscores the systemic risks posed by excessive alcohol consumption and emphasizes that reducing alcohol consumption is the safest strategy to mitigate the broad spectrum of alcohol-related health complications.

## Glossary

ACS: acetyl-CoA synthetase
ADH: alcohol dehydrogenase
AFL: alcoholic fatty liver
ALD: alcohol-related liver disease
ALDH: aldehyde dehydrogenase
acetyl-CoA: acetyl coenzyme A
CCL2: chemokine (C-C motif) ligand 2
CYP2E1: cytochrome P450 2E1
FAEE: fatty acid ethyl esters
FFA: free fatty acid
GI: gastrointestinal
IL-1β: interleukin-1β
IL-6: interleukin-6
LDH: lactate dehydrogenase
LPS: lipopolysaccharide
mtDNA: mitochondrial DNA
NAMPT: nicotinamide phosphoribosyltransferase
**NAD** ^**+**^: nicotinamide adenine dinucleotide
NADH: a reduced form of nicotinamide adenine dinucleotide
NADPH: nicotinamide adenine dinucleotide phosphate
NF-κB: nuclear factor kappa-light-chain-enhancer of activated B cells
NLRP3: NOD-like receptor family pyrin domain containing 3
PPAR: peroxisome proliferator-activated receptor
RNS: reactive nitrogen species
ROS: reactive oxygen species
SCFA: short-chain fatty acid
SIRT1: sirtuin 1
TCA: tricarboxylic acid
T2DM: type 2 diabetes mellitus
TLR4: toll-like receptor 4
TNF: tumor necrosis factor
**UPR** ^**mt**^: mitochondrial unfolded protein response.
